# Victims and Perpetrators of Intimate Partner Violence Among Sexually Active Youth in a Community With a High HIV Prevalence in Western Kenya

**DOI:** 10.24248/EAHRJ-D-18-00019

**Published:** 2018-11-23

**Authors:** Barbara Burmen, George Olilo, Ester M Makanga

**Affiliations:** a Centre for Global Health Research, Kenya Medical Research Institute, Kisumu, Kenya

## Abstract

**Background:**

Physical intimate partner violence (IPV) is an important risk factor for sexually transmitted infections, including HIV. We set out to determine the prevalence and correlates of IPV among youth aged 15 to 24 years – in a community with a high HIV prevalence – with a view to recommending strategies to address IPV.

**Methods:**

We analysed data from an HIV seroprevalence survey, which included participants aged 13 years and above and was conducted between November 2012 and December 2014 in Gem Subcounty, Siaya County, Western Kenya. Participants between 15 and 24 years old (youth) were described as “perpetrators of IPV” if they had done anything to physically hurt their sexual partners in the previous year and as “victims of IPV” if they had been physically hurt by a sexual partner in the same timeframe. Logistic regression was used to determine factors associated with being either a victim or perpetrator of IPV.

**Results:**

Of 1,957 participants included in the analysis, 142 (7%) were victims of IPV, and 77 (4%) were perpetrators of IPV. Victims were likely to be women (adjusted odds ratio [AOR] 7.9; 95% CI, 3.6 to 17.5), in a relationship or married (AOR 3.1; 95% CI, 1.8 to 5.4), and to have had multiple lifetime sexual partners. Victims of IPV were also more likely than not to have been subjected to sexual violence in the past (AOR 1.9; 95% CI, 1.0 to 3.4) or recently (AOR 3.9; 95% CI, 2.2 to 6.8). Perpetrators were likely to be men (AOR 2.1; 95% CI, 1.2 to 3.7), with 5 or more lifetime sexual partners (AOR 2.8; 95% CI, 1.3 to 6.3), and to have committed sexual violence recently (AOR 2.9; 95% CI, 1.1 to 7.7).

**Conclusion:**

There was a high prevalence of IPV among sexually active youth in this rural community. Study participants were recurrent victims or perpetrators and reported behaviours that put them at risk of HIV acquisition. Health programmes should screen for IPV victims and perpetrators using identified characteristics. Existing policies regarding gender-based violence should be enforced, and future research should focus on the impact of IPV prevention programmes.

## INTRODUCTION

In 2017, 1.8 million new HIV infections and 36.9 million people living with HIV were reported worldwide. Two-thirds of those new infections and 25.7 million of the people living with HIV were from in sub-Saharan Africa.^1^ Youth aged 15 to 24 years accounted for 42% of new HIV infections in people aged 15 years and older. In 2012, globally, young women aged 15 to 24 years had HIV infection rates twice as high as young men and accounted for 22% of all new HIV infections, including 31% of new infections in sub-Saharan Africa.^2^

Globally, 10% to 69% of women report having been assaulted by an intimate male partner.^3^ Physical intimate partner violence (IPV) is an important risk factor for sexually transmitted infection and HIV transmission.^4,5^ Research has shown that interrelationships between IPV and other forms of violence also increase the risk of HIV transmission.^6^ In Zambia, among ever-married women, those who had experienced any form of IPV were twice as likely to be HIV-positive compared with those who had not experienced IPV.^7^ IPV has also been linked to poor HIV testing and antiretroviral therapy uptake^8^ as well as poor antiretroviral therapy outcomes.^9^

In 2015, HIV acquisition among youth aged 15 to 24 – who formed 20% of the population – constituted more than half of all new HIV infections and one-fifth of people living with HIV in Kenya.^10^ Low HIV testing uptake and linkage to care rates have been shown among children, adolescents, and young adults in Kenya.^11^ Combating IPV is likely to reduce the spread of HIV and improve the uptake of HIV health services. The general strategy to combat IPV can be either preventative or therapeutic^6^; however, this requires identifying actual or potential victims and perpetrators of IPV.

We set out to determine the prevalence of IPV among youth within the Kenya Medical Research Institute and U.S. Centers for Disease Control and Prevention (KEMRI/CDC) Health and Demographic Surveillance Area (HDSA). We also aimed to determine factors that correlate with IPV, with a view to recommending strategies to prevent and address IPV in the Western Region of Kenya, an area with a high HIV burden.

## METHODS

### Study Design and Setting

KEMRI/CDC's research and public health collaboration conducted a cross-sectional survey within its HDSA in Gem Subcounty, Siaya County, Western Kenya, between January 2013 and February 2014. The KEMRI/CDC HDSA has a population of approximately 218,376 people living in 70,505 households within 3 regions: 61,707 in Asembo, 78,874 in Gem, and 77,795 in Karemo. As there had been minimal research and intervention activities rolled out in Gem, it was an ideal community for assessing the effects of new interventions. Gem's population is culturally homogeneous and survives on subsistence farming and fishing; over 95% are members of the Luo tribe, and 50% are younger than 13 years of age. Detailed descriptions of the study design and methods are described in our other papers.^12,13^ The survey aimed to evaluate HIV risk behaviours, HIV serostatus, and HIV prevention interventions.

### Study Population

The study population in the main survey included all persons aged 13 years of age or older, who lived within the selected compounds, had spent the previous night in the designated households, and consented to participate in the study. Individuals who did not consent to participate were excluded. We restricted our analysis to youth aged 15 to 24 years,^14^ who had been sexually active in the past year, and had answered questions about ever having been a victim or perpetrator of IPV.

### Sampling

Of 14,501 compounds registered in Gem in 2010, we randomly selected 6,000, partly by community sampling (750 households) via a participatory community event and partly by computer-generated statistical sampling (5,250 households) conducted by the HDSA data team. The study statistician randomly sampled the remaining compounds using a computer. Details of these sampling methods are described by Phillips-Howard et al.^15^

### Data Collection

For all participants, interview topics included participant demographics, sexual behaviour, and utilisation of HIV health services. From data collected during the survey, we extracted a database of persons aged 15 to 24 to address our research questions.

### Outcome Variable Definitions

For this analysis, we adapted the United Nations definition of IPV to include only ‘physical harm from a current or former intimate partner’.^16^ Participants were described as “victims of IPV” if they answered “yes” to the question, ‘Has any of your sexual partners, in the last year, hit, slapped, kicked, or done anything else to hurt you physically?’ Participants were identified as “perpetrators of IPV” if they answered in the affirmative to the question, ‘Have you, in the last year, hit, slapped, kicked, or done anything else to physically hurt any of your sexual partners?’

### Definitions of Independent Variables of Interest

A sexual partner was described as a “recent sex partner” if he or she had been a sexual partner of the interviewee within 1 year preceding the interview. Study participants were described as “single” if they reported not having a romantic or cohabiting partner or spouse at the time of interview, including if they were separated or widowed. “In a relationship or married” was defined as participants who were in a monogamous or polygamous relationship, cohabiting, or married.

Participants were characterised as having ever been subjected to “sexual violence in the past” if they answered “yes” to the question, ‘Have you ever been forced to have sex?’ Participants who had been subjected to “sexual violence recently” were those who answered in the affirmative to the question, ‘In the last 12 months, has partner X forced you to have sex?’ Depending on the interviewee's sexual history, “partner X” referred to any of the interviewee's 3 most recent sexual partners in the year preceding the interview. Conversely, participants had committed “sexual violence recently” if they answered affirmatively to the question, ‘In the last 12 months, have you forced any of your sexual partners to have sex?’

Participants were described as ever having experienced “a condom error” with a recent sexual partner if they answered “yes” to the any of the following questions: ‘While using condoms with partner X, did you ever put on the condom after you had already started having sexual intercourse?’, ‘Did you ever take off the condom before you were finished having sexual intercourse?’, ‘Did the condom you were using ever slip off during sex or while pulling out?’, or ‘Did the condom you were using ever break or leak during sex or while pulling out?’

### Data Analysis

Proportions were used to describe participant characteristics. Chi-square or Fisher's exact tests were used to compare participants according to their history of having been subjected to or having perpetrated IPV. Logistic regression was used to determine factors associated with being either victims or perpetrators of IPV. Variables that attained a *P* value less than .2 in the univariate analysis were included in the multivariate logistic regression model. Using backward elimination criteria, variables that had a *P* value less than .1 were retained in the multivariate model. Variables that had a *P* value less than .05 were considered significant. Crude odds ratios, which explained the relationship between a given variable and the outcome, were reported. Adjusted odds ratios (AORs), which included the influence of other variables on the outcome, were also reported. All estimates were reported with 95% confidence intervals (CIs).^17^ Analysis was done using Statistical Analysis Software (SAS), version 9.2 (SAS Institute Inc., Cary, NC, USA).

### Ethical Approval

Permission to conduct this study was granted by the Kenya Medical Research Institute Ethics Review Committee (SSC No. 1801).

## RESULTS

### Participant Selection

Of 14,116 interviewees, 5,225 (37%) were youths, 1,992 (38%) of whom had been sexually active in the previous 12 months. Among these, 1,957 (98%) answered questions regarding having ever been subjected to or having ever perpetrated IPV (**Figure**).

**FIGURE:**
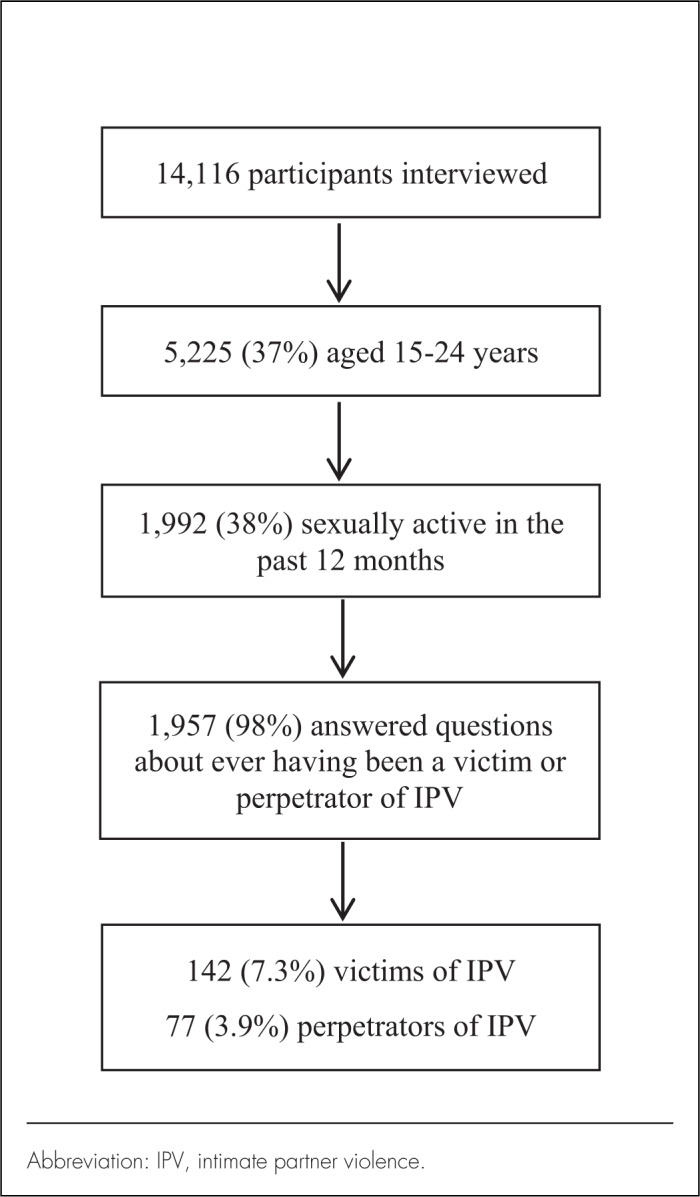
Participant Selection

### Participant Characteristics

Of the 1,957 participants included in the analysis, the majority were aged 19 to 22 years (n=1,002; 51%), female (n=1,174, 60%), single (n=993, 51%), had primary or below primary-level education (n=1,349, 69%), and engaged in some form of employment (n=1,054, 54%).

At the time of their respective interviews, participants frequently reported having had 2 lifetime sexual partners (n=563, 31%) and 1 recent sexual partner (n=1,687, 86%). Some participants reported having a sexual partner who had other concurrent sexual partners (n=267, 14%) or had newly acquired other sexual partners (n=233, 12%). Regarding experience with sexual violence, 131 (7%) participants reported that they had been subjected to sexual violence recently, 134 (7%) had been subjected to sexual violence in the past, and 48 (3%) had recently committed sexual violence.

The majority of participants had previously used a condom during sexual intercourse (n=1,239, 63%) or had at some point asked a sexual partner to use a condom (n=996, 51%). Conversely, less than half (n=842, 43%) had used condoms during their most recent sexual intercourse, and 205 (11%) reported having experienced condom errors with a recent sexual partner (**Table 1**).

**TABLE 1.** Characteristics of Youth Interviewed in Gem, Siaya County, Western Kenya, 2013–2014 (N=1,957)

Characteristics	n (%)
**Age group, years**	
15–18	410 (21)
19–22	1,002 (51)
23–24	546 (28)
**Gender**	
Male	784 (40)
Female	1,174 (60)
**Marital status**	
Single^a^	993 (51)
In a relationship or married	964 (49)
**Education level**	
Primary or below	1,349 (69)
Above primary	608 (31)
**Occupation**	
Employed	1,054 (54)
Unemployed	905 (46)
**Lifetime number of sex partners**^b^	
1	449 (25)
2	563 (31)
3–4	548 (30)
5 and above	264 (14)
**Number of sex partners in the last 12 months**	
1	1,687 (86)
2 and above	268 (14)
**Primary sexual partner has other partners**	
Yes	267 (14)
No	1,692 (86)
**Primary sexual partner recently acquired a new partner**	
Yes	233 (12)
No	1,726 (88)
**Subjected to sexual violence in the past**^c^	
Yes	134 (7)
No	1,825 (93)
**Subjected to sexual violence recently**^d^	
Yes	131 (7)
No	1,828 (93)
**Committed sexual violence recently**^e^	
Yes	48 (3)
No	1,911 (97)
**Ever used a condom**	
Yes	1,239 (63)
No	720 (34)
**Ever asked partner to use a condom**	
Yes	996 (51)
No	963 (49)
**Used a condom during last sexual intercourse**	
Yes	842 (43)
No	1,117 (57)
**Experienced condom error in the past 12 months**	
Yes	205 (11)
No	1,754 (89)
**Ever consumed alcohol before sex or been drunk during sex**	
Yes	67 (3)
No	1,892 (97)
**Partner ever consumed alcohol before sex or been drunk during sex**	
Yes	77 (4)
No	1,882 (97)
**Used drugs or mind-altering substances in the past year**	
Yes	45 (2)
No	1,914 (98)
**Partner used drugs or mind-altering substances in the past year**	
Yes	51 (3)
No	1,908 (97)

a Includes 32 participants who were either divorced or widowed.

b Responses missing for 135 respondents.

c Participants were characterised as having ever experienced ‘sexual violence in the past’ if they answered “yes” to the question, ‘Have you ever been forced to have sex?’

d Participants were characterised as having been subjected to ‘sexual violence recently’ if they answered in the affirmative to the question, ‘In the last 12 months has partner X forced you to have sex?’

e Participants were characterised as having ever committed sexual violence if they answered “yes” to the question, ‘In the last 12 months have you forced any of your sex partners to have sex?’

In the year preceding the survey, a minority of participants reported that they themselves (n=45, 2%) or their sexual partners (n=51, 3%) had taken drugs or mind-altering substances. Few participants reported having consumed alcohol before sex or being drunk during sex (n=67, 3%) or having had sexual partners who had consumed alcohol before or been drunk during sexual intercourse (n=77, 4%) (**Table 1**).

### Victims of Intimate Partner Violence

Of the 1,957 participants, 142 (7%) reported having ever been victims of IPV. Of the 142 past victims of IPV, 29 (22%) reported having been subjected to sexual violence within the 12 months before being interviewed, and 40 (31%) said they had been subjected to sexual violence recently (AOR 3.9; 95% CI, 2.2 to 6.8; *P*<.01). Victims of IPV were more likely to be female (n=131, 92%) than male (n=11, 8%; AOR 7.9; 95% CI, 3.6 to 17.5; *P*<.01), and to be in a relationship or married (n=118, 83%) than single (n=24, 17%; AOR 3.1; 95% CI, 1.8 to 5.4; *P*<.01). Overall, 11% of females were victims of IPV, compared to 1% of males (**Table 2**).

**TABLE 2.** Factors Associated With Being a Victim of Intimate Partner Violence^a^ in the Past Year Among Youths in Western Kenya, 2013–2014^b^

Characteristics	Victims of IPV^c^ n/Row Total (%)	Crude Odds Ratio^d^ (95% CI)	*P* Value	Adjusted Odds Ratio^e^ (95% CI)	*P* Value
**Age group, years**					
15–18	19/410 (5)	Ref	.03		
19–22	73/1,001 (7)	1.6 (0.9–2.7)			
23–24	50/546 (9)	2.1 (1.2–3.6)			
**Gender**					
Male	11/784 (1)	Ref	<.01	Ref	<.01
Female	131/1,173 (11)	8.8 (4.7–16.4)		7.9 (3.6–17.5)	
**Marital status**					
Single^f^	24/969 (2)	Ref	<.01	Ref	<.01
In a relationship or married	118/964 (12)	5.6 (3.6–8.8)		3.1 (1.8–5.4)	
**Education level**					
Primary or below	119/1,349 (9)	2.5 (1.6–3.9)	<.01		
Above primary	23/608 (4)	Ref			
**Occupation**					
Employed	90/1,053 (9)	1.5 (1.1–2.2)	.02		
Unemployed	52/904 (6)	Ref			
**Lifetime number of sex partners**^g^					
1	17/432 (4)	Ref	<.01	Ref	<.01
2	34/563 (6)	1.6 (0.9–2.9)		1.2 (1.9–8.4)	
3–4	59/546 (11)	3.1 (1.7–5.4)		2.1 (1.2–3.9)	
5 and above	25/264 (9)	2.7 (1.4–5.0)		4.0 (1.9–8.4)	
**Number of sex partners in the last 12 months**					
1	129/1,685 (8)	1.6 (0.9–2.9)	.10		
2 and above	13/268 (5)	Ref			
**Primary sexual partner has other partners**					
Yes	32/233 (14)	2.3 (1.5–3.6)	<.01		
No	110/1,724 (6)	Ref			
**Primary sexual partner recently acquired a new partner**					
Yes	39/267 (15)	2.6 (1.8–3.9)	<.01	2.7 (1.7–4.2)	<.01
No	103/1,690 (6)	Ref		Ref	
**Subjected to sexual violence in the past**^h^					
Yes	29/105 (22)	4.2 (2.7–6.6)	<.01	1.9 (1.0–3.4)	<.01
No	113/1,823 (6)	Ref		Ref	
**Subjected to sexual violence recently**^i^					
Yes	40/131 (31)	7.4 (4.9–11.3)	<.01	3.9 (2.2–6.8)	<.01
No	102/1,724 (6)	Ref		Ref	
**Committed sexual violence recently**^j^					
Yes	9/48 (19)	3.1 (1.5–6.5)	<.01		
No	133/1,909 (7)	Ref			
**Ever used a condom**					
Yes	73/1,238 (6)	Ref	<.01		
No	69/719 (10)	1.7 (1.2–2.4)			
**Ever asked partner to use a condom**					
Yes	70/995 (7)	0.9 (0.7–1.3)	.70		
No	72/962 (7)	Ref			
**Used a condom during last sexual intercourse**					
Yes	37/842 (4)	Ref	<0.01		
No	105/1,115 (9)	2.2 (1.5–3.3)			
**Experienced condom error reported in the last 3 months**					
Yes	15/204 (7)	1.0 (0.6–1.8)	.90		
No	127/1,753 (7)	Ref			
**Ever consumed alcohol before or during sex or been drunk during sex**					
Yes	9/67 (13)	2.0 (0.9–4.2)	.05		
No	133/1,890 (7)	Ref			
**Partner ever consumed alcohol before or during sex or been drunk during sex**					
Yes	21/77 (27)	5.5 (3.1–9.3)	<.01		
No	121/1,880 (6)	Ref			
**Used drugs or mind-altering substances in the past year**					
Yes	1.45 (2)	Ref	.20		
No	141/1,912 (7)	0.3 (0.03–2.1)			
**Partner used drugs or mind-altering substances in the past year**					
Yes	15/51 (29)	5.8 (3.1–10.9)	<.01		
No	127/1,906 (7)	Ref			

a Participants were described as “victims of IPV” if they answered “yes” to the question, ‘Has any of your sexual partners, in the last year hit, slapped, kicked, or done anything else to hurt you physically?’

b Responses are missing for 2 participants who did not answer questions about ever having experienced physical IPV.

c There were 142 (7%) victims of IPV; the numerators in this column are the number of victims of IPV who fulfilled the criteria described in the respective rows, and the denominators are the total number of participants who fulfilled the criteria mentioned in each row.

d Crude odds ratios refer to the odds of an outcome given the response status of a particular variable.

e Adjusted odds ratios are crude odds ratios adjusted after considering the influence of all other variables.

f Includes 32 participants who were either divorced or widowed.

g Responses missing for 135 participants.

h Participants were characterised as having ever been subjected to “sexual violence in the past” if they answered “yes” to the question, ‘Have you ever been forced to have sex?’

i Participants were characterised as having been subjected to “sexual violence recently” if they answered in the affirmative to the question, ‘In the last 12 months has partner X forced you to have sex?’

j Participants were characterised as having committed “sexual violence recently” if they answered “yes” to the question, ‘In the last 12 months have you forced any of your sex partners to have sex?’

Abbreviations: CI, confidence interval; IPV, intimate partner violence.

According to 135 available records, victims of IPV were also more likely to have had either 2 (n=34, 25%; AOR 1.2; 95% CI, 1.9 to 8.4), 3 to 4 (n=59, 43%; AOR 2.1; 95% CI, 1.2 to 3.9), or 5 or more (n=25, 19%; AOR 4.0; 95% CI, 1.9 to 8.4) lifetime sexual partners than to have had 1 (n=17, 13%) lifetime sexual partner (*P*<.01). Furthermore, among victims of IPV, 32 (23%) had primary sexual partners who had additional concurrent partners, and 39 (27%) had primary partners who had recently acquired new sexual partners. There were 29 (20%) victims of IPV who reported having been subjected to sexual violence in the past, compared with 113 (80%) who had not been subjected to sexual violence more than 12 months prior (AOR 1.9; 95% CI, 1.0 to 3.4; *P*<.01).

### Perpetrators of Intimate Partner Violence

Of the 1,957 participants, 76 (4%) reported having ever been perpetrators of IPV. Perpetrators of IPV were more likely to be male (n=52, 68%) than female (n=24, 32%; AOR 2.1; 95% CI, 1.2 to 3.7; *P*<.01). Among 66 available records, perpetrators of IPV were more likely to have had 5 or more (n=27, 41%) lifetime sexual partners than 1 (n=10, 15%; AOR 2.8; 95% CI, 1.3 to 6.3, *P*<.01) lifetime sexual partner. Among the 76 participants who identified themselves as perpetrators of IPV in the previous year, 7 (9%) also reported committing sexual violence within the same period, compared to 69 (91%) who reported that they had not recently committed sexual violence (AOR 2.9; 95% CI, 1.1 to 7.7; *P*=.02). Overall, 7% of males and 2% of females identified themselves as perpetrators of IPV (**Table 3**).

**TABLE 3.** Factors Associated With Being a Perpetrator of Intimate Partner Violence^a^ in the Past Year Among Youths in Western Kenya, 2013–2014^b^

Characteristics	Perpetrators of IPV^c^ n/Row Total (%)	Crude Odds Ratio^d^ (95% CI)	*P* Value	Adjusted Odds Ratio^e^ (95% CI)	*P* Value
**Age group, years**					
15–18	12/410 (3)	Ref	.50		
19–22	41/1,001 (4)	1.4 (0.7–2.7)			
23–24	23/546 (4)	1.5 (0.7–2.9)			
**Gender**					
Male	52/784 (7)	3.4 (2.1–5.6)	<.01	2.1 (1.2–3.7)	.01
Female	24/1,149 (2)	Ref		Ref	
**Marital status**					
Single^f^	39/993 (4)		.92		
In a relationship or married	37/964 (4)	Ref			
**Education level**					
Primary or below	50/1,349 (4)	0.8 (0.5–1.4)	.50		
Above primary	26/608 (4)	Ref			
**Occupation**					
Employed	51/1,053 (5)	1.8 (1.1–2.9)	.02		
Unemployed	25/904 (3)	Ref			
**Lifetime number of sex partners**^g^					
1	10/449 (2)	Ref	<.01	Ref	<.01
2	9/563 (2)	0.7 (0.3–1.8)		0.7 (0.3-1.6)	
3–4	20/546 (4)	1.7 (0.8–3.6)		1.3 (0.6–2.9)	
5 and above	27/264 (10)	5.0 (2.4–10.5)		2.8 (1.3–6.3)	
**Number of sex partners in the last 12 months**					
1	50/1,685 (3)	Ref	<.01		
2 and above	25/268 (9)	3.4 (2.0–5.5)			
**Primary sexual partner has other partners**					
Yes	16/233 (7)	2.0 (1.2–3.6)	.01		
No	60/1,724 (4)				
**Primary sexual partner has a new partner**					
Yes	21/267 (8)	2.5 (1.5–4.3)	<.01		
No	55/1,690 (3)	Ref			
**Subjected to sexual violence in the past**^h^					
Yes	8/134 (6)	1.6 (0.8–3.5)	.20		
No	68/1,823 (4)	Ref			
**Subjected to sexual violence recently**^i^					
Yes	9/131 (7)	1.9 (0.9–3.9)	.07		
No	67/1,826 (4)	Ref			
**Committed sexual violence recently**^j^					
Yes	7/48 (15)	4.6 (1.9–10.5)	<.01	2.9 (1.1–7.7)	.02
No	69/1,909 (4)	Ref		Ref	
**Ever used a condom**					
Yes	54/1,238 (4)	1.4 (0.9–2.4)	.20		
No	22/719 (3)	Ref			
**Ever asked partner to use a condom**					
Yes	49/995 (5)	1.8 (1.1–2.9)	.02		
No	27/962 (3)	Ref			
**Used a condom during last sexual intercourse**					
Yes	35/842 (4)	1.1 (0.7–1.8)	.80		
No	41/1,115 (4)				
**Experienced condom error in the last 3 months**					
Yes	17/2,014 (8)	2.6 (1.5–4.6)	<.01		
No	59/1,753 (3)	Ref			
**Ever consumed alcohol before or during sex or been drunk during sex**					
Yes	4/67 (6)	1.6 (0.6–4.5)	.40		
No	72/1,890 (4)	Ref			
**Partner ever consumed alcohol before or during sex or been drunk during sex**					
Yes	5/77 (6)	1.8 (0.7–4.5)	.20		
No	71/1,880 (4)	Ref			
**Used drugs or mind-altering substances in the past year**					
Yes	5/45 (11)	3.2 (1.2–8.5)	.01		
No	71/1,912 (4)	Ref			
**Partner used drugs or mind-altering substances in the past year**					
Yes	1/51 (2)	Ref	.50		
No	75/1,906 (4)	0.5 (0.1–3.6)			

a Participants were described as “perpetrators of IPV” if they answered in the affirmative to the question, ‘Have you, in the last year, hit, slapped, kicked, or done anything else to physically hurt any of your sexual partners?’

b Responses missing for 2 participants who did not answer questions about ever having perpetrated physical IPV.

c There were 77 (4%) perpetrators of IPV; the numerators in this column are the number of perpetrators of IPV who fulfilled the criteria described in the respective rows, and the denominators are the total number of participants who fulfilled the criteria mentioned in each row.

d Crude odds ratios refer to the odds of an outcome given the response status of a particular variable.

e Adjusted odds ratios are crude odds ratios adjusted after considering the influence of all other variables.

f Includes 32 participants who were either divorced or widowed.

g Responses missing for 135 participants.

h Participants were characterised as having ever been subjected to “sexual violence in the past” if they answered “yes” to the question, ‘Have you ever been forced to have sex?’

i Participants were characterised as having been subjected to “sexual violence recently” if they answered in the affirmative to the question, ‘In the last 12 months has partner X forced you to have sex?’

j Participants were characterised as having committed “sexual violence recently” if they answered “yes” to the question, ‘In the last 12 months have you forced any of your sex partners to have sex?’

Abbreviations: CI, confidence interval; IPV, intimate partner violence.

## DISCUSSION

Among the 1,957 sexually active youths in our study population, 7% were victims and 4% were perpetrators of IPV. The prevalence of IPV was lower than what was found in a 2005 survey conducted in 10 countries by the World Health Organization. That study revealed that 13% to 61% of women who had ever been in an intimate partnership had been subjected to physical violence by a partner.^6^ A Kenyan national survey, conducted in 2014, found that one-fifth of all women aged 15 years and older had experienced some form of physical violence.^18^ It is important to highlight that our study sample was limited to youth aged 15 to 24 years, as opposed to other studies that may have included broader age ranges. Nevertheless, younger individuals have been shown to have higher rates of IPV,^19^ and intervening at this stage is therefore likely to reduce the chances of lifetime victimisation and perpetration, which increase the risk of HIV transmission.^5^

While being interviewed, victims of IPV – who were mostly female and of low educational status – were likely to be in a current relationship with a partner whose abuse qualified the partnered individuals to be a victim and perpetrator of IPV, according to the study definitions. In the 2014 Kenya Demographic and Health Survey, more than half (57%) of women who had ever experienced physical violence stated that the perpetrator was the current spouse.^19^ Similar results have been reported in India and South Africa.^20,21^ This could be related to a high level of economic dependency by women on men,^16^ which may make many women reluctant to report IPV.^22^ In Uganda, a decrease in IPV has been associated with the empowerment of women, providing evidence to support the importance of education and delayed partnering for young women.^19,23^

Our assessment corroborated the presumption that perpetrators are usually male. In the literature, the perpetrators of IPV are typically men living in communities where violence is routinely used to resolve problems. Perpetrators in such communities often feel their male identity being challenged by factors related to poverty.^16^ Although our study found higher rates of IPV perpetration among males, other sources report similar rates of IPV perpetration by men and women, with women less likely than men to commit severe violence. IPV inflicted by women on men is likely to be underreported due to social desirability bias, and limited information exists in the literature about male victims of IPV.^19^

Many victims and perpetrators reported having had more than 1 lifetime sexual partner and having been subjected to or having committed IPV over a duration that extends earlier than the preceding 12 months considered in the study definition of IPV. In India^20^ and South Africa,^21^ women who reported IPV were more likely to have been abused in the past. As both IPV^5^ and multiple sex partners^24^ are risk factors for HIV transmission, it is important to screen for IPV and provide interventions that may mitigate future occurrences. This has been the basis for second responder programmes in the United States.^25^

While both victims and perpetrators reported several lifetime partners, only victims reported that their partners had concurrent sexual partners. This finding is similar to reports from Togo and South Africa, where HIV-infected women who were victims of IPV also reported that their partners had multiple partners.^26,27^ This may be related to a common double standard regarding the sexual behaviour of men and women: while women with multiple sex partners are viewed as promiscuous, men receive praise for their sexual experiences with multiple partners. In South Africa, teenage girls stated that victims have multiple sexual partners to find solace or as a form of resistance.^28^ Conversely, perpetrators may seek multiple sexual partners as a form of male dominance.^29^

### Limitations

This study's limitations included the sole focus on physical violence without consideration of sexual or psychological forms of IPV. This analysis was also limited by recall and respondent biases; participants may have felt embarrassed to admit to the interviewers that they had been subjected to or had perpetrated IPV. For these reasons, our estimates of the burden of IPV in this population may be underestimated.

## RECOMMENDATIONS AND CONCLUSION

There was a high prevalence of IPV among sexually active youth in this rural community. Study participants were recurrent victims or perpetrators and reported behaviours that put them at risk of HIV acquisition. Victims and perpetrators also possessed characteristics that could be used by screening programmes to identify and target them for specific interventions.

There is a need to reduce gender inequality and to enhance the livelihoods of young women via upstream interventions. The Ministry of Health in Kenya provides structural prevention approaches to bolster resilience among women and girls through behavioural interventions, including evidence-based behavioural HIV prevention strategies to equip young girls with the skills to negotiate safe sex. The Ministry also conducts targeted sensitisation about IPV, for example, in conjunction with the United States Agency for International Development and the Determined, Resilient, Educated, AIDS-free, Mentored, and Safe (DREAMS) mentorship programme for adolescents and young women.^30^

Downstream interventions to address IPV could include screening and provision of gender-based violence and recovery centres and services. Health-care workers should also screen for other forms of IPV among women presenting to health-care facilities with physical injuries, depression symptoms, and miscarriages, or for routine care.^31^ The potential benefits of health-care screening programmes rely on client expectations of compassionate, nonjudgemental, and effective care delivery from health-care providers.^32^ If this is accomplished, health workers can provide appropriate referrals to further reduce exposure to IPV and its consequences.^32^ Fragmented care has been identified as a barrier to effective management of IPV. Because gender-based violence services are not routinely offered as part of standard care, victims of IPV are often lost along the referral cascade. Gender-based violence services should, therefore, be diversified to include legal services and professional counselling in addition to health services.^22^ There should also be clear laws related to IPV to enhance legal reporting and clear guidelines on the management of all forms of IPV – not just physical violence.^33^

Health-care programmes should also publicise the availability of gender-based violence and recovery services to both health-care providers and communities to encourage reporting of IPV. Information campaigns have been shown to enhance clients' perspectives on the availability of facilities to assist victims of IPV.^34^ Furthermore, community mobilisation of gender-based violence service campaigns in Kisumu County, Kenya, led to an increase in the number of cases that were seen at the gender-based violence centre there (HIV Prevention Coordinator, KEMRI Centre for Global Health Research, personal communication, 12 June 2014).

Several integrated approaches can be used to prevent IPV. Upstream approaches include legislation to deter potential offenders and punish reported offenders.^33^ Policy makers should enforce existing legislation, such as the Sexual Offences Act in the Constitution of Kenya, which will deter potential perpetrators of IPV and reprimand identified perpetrators.^35^ Policy makers also ought to address other determinants of IPV, including poverty, drug abuse, economic dependency on men by victims of IPV, and societal norms. The provision of life skills training to address known risk factors for perpetrating IPV – such as alcoholism and unemployment – and the use of renowned male role models as ambassadors against IPV, would also contribute to preventing IPV.^6,36^

Upon identification of IPV perpetrators, downstream interventions should include needs and risk assessments of the perpetrators and their immediate family environments. The findings of such evaluations should then be incorporated into programmes to motivate the perpetrators to change their maladaptive behaviours.^37^ Perpetrators should also enrol in batterer intervention programmes.^38^ In the United States, a second responder intervention focused on assessed police outcomes of previous perpetrators randomised to an intervention group that provided risk profiling, interventions based on men's criminogenic triggers, and responsivity. Compared to a control group, men in the intervention group reported lower rates of domestic violence.^25^ This exemplified the effectiveness of interventions that are tailored to the learning styles and motivations of intervention participants. As more than one-tenth of perpetrators reported having ever committed sexual violence recently in our evaluation, there is a need for targeted interventions. Communities should be sensitised to the dangers of IPV and other means of improving communication and conflict resolution within partnerships.^30^

In 2010, voluntary HIV counselling and testing centres were proposed as ideal places to identify victims of IPV, because these centres offer opportunities to discuss risky sexual behaviour and HIV prevention.^24^ However, without adequate skills to discuss gender inequality issues, lay counsellors conducting screening for IPV were unable to offer solutions.^39^ In response to this, in 2014, a couples HIV risk-reduction programme implemented by South African community health centre workers achieved IPV reduction over a period of 1 year.^40^ Couples counselling and testing would, therefore, be an ideal setting to broach the issue of IPV in the context of HIV risk reduction counselling and HIV testing.

Further research is required to assess the proportion of IPV incidents that are reported, quantify the burden of IPV (including other forms of violence in the definition of IPV), determine the motivating and contributing factors behind IPV, and assess the impact of any programmes that address IPV perpetrators.
